# Chronic obstructive pulmonary disease mortality and prevalence: the associations with smoking and poverty—a BOLD analysis

**DOI:** 10.1136/thoraxjnl-2013-204460

**Published:** 2013-12-18

**Authors:** Peter Burney, Anamika Jithoo, Bernet Kato, Christer Janson, David Mannino, Ewa Niżankowska-Mogilnicka, Michael Studnicka, Wan Tan, Eric Bateman, Ali Koçabas, William M Vollmer, Thorarrin Gislason, Guy Marks, Parvaiz A Koul, Imed Harrabi, Louisa Gnatiuc, Sonia Buist, for the Burden of Obstructive Lung Disease (BOLD) Study

**Affiliations:** 1 National Heart & Lung Institute, Imperial College, London, UK; 2 Department of Medical Sciences: Respiratory Medicine & Allergology, Uppsala University, Uppsala, Sweden; 3 University of Kentucky, Lexington, Kentucky, USA; 4 Jagiellonian University School of Medicine, Cracow, Poland; 5 Department of Pulmonary Medicine, Paracelsus Medical University, Salzburg, Austria; 6 University of British Columbia, Vancouver, British Columbia, Canada; 7 University of Cape Town Lung Institute, Cape Town, South Africa; 8 Cukurova University School of Medicine, Adana, Turkey; 9 Kaiser Permanente Center for Health Research, Portland, Oregon, USA; 10 Landspitali University Hospital, Reykjavik, Iceland; 11 Woolcock Institute of Medical Research, Sydney, Australia; 12 Sher-i-Kashmir Institute of Medical Sciences, Srinagar, Jammu and Kashmir, India; 13 Faculté de Médecine, Sousse, Tunisia; 14 Oregon Health & Sciences University, Portland, Oregon, USA

**Keywords:** COPD epidemiology, Tobacco and the lung, Lung Physiology

## Abstract

**Background:**

Chronic obstructive pulmonary disease (COPD) is a commonly reported cause of death and associated with smoking. However, COPD mortality is high in poor countries with low smoking rates. Spirometric restriction predicts mortality better than airflow obstruction, suggesting that the prevalence of restriction could explain mortality rates attributed to COPD. We have studied associations between mortality from COPD and low lung function, and between both lung function and death rates and cigarette consumption and gross national income per capita (GNI).

**Methods:**

National COPD mortality rates were regressed against the prevalence of airflow obstruction and spirometric restriction in 22 Burden of Obstructive Lung Disease (BOLD) study sites and against GNI, and national smoking prevalence. The prevalence of airflow obstruction and spirometric restriction in the BOLD sites were regressed against GNI and mean pack years smoked.

**Results:**

National COPD mortality rates were more strongly associated with spirometric restriction in the BOLD sites (<60 years: men r_s_=0.73, p=0.0001; women r_s_=0.90, p<0.0001; 60+ years: men r_s_=0.63, p=0.0022; women r_s_=0.37, p=0.1) than obstruction (<60 years: men r_s_=0.28, p=0.20; women r_s_=0.17, p<0.46; 60+ years: men r_s_=0.28, p=0.23; women r_s_=0.22, p=0.33). Obstruction increased with mean pack years smoked, but COPD mortality fell with increased cigarette consumption and rose rapidly as GNI fell below US$15 000. Prevalence of restriction was not associated with smoking but also increased rapidly as GNI fell below US$15 000.

**Conclusions:**

Smoking remains the single most important cause of obstruction but a high prevalence of restriction associated with poverty could explain the high ‘COPD’ mortality in poor countries.

**Key messages**

**What is the key question?**

- What is the relation between the global distribution of chronic obstructive pulmonary disease (COPD) mortality, the prevalence of abnormal lung function, smoking and poverty?

**What is the bottom line?**

- Smoking prevalence correlates with airflow obstruction, but not with mortality from COPD; COPD mortality is associated with low vital capacity; COPD mortality and low vital capacity are associated with poverty.

**Why read on?**

- Between 1990 and 2010, COPD rose from the fourth to the third most common cause of death globally. Adequate understanding of the distribution of COPD mortality is the key to finding an adequate response.

## Introduction

Chronic obstructive pulmonary disease (COPD) is now the third most common cause of death in the world.^1^ COPD is defined in terms of airflow obstruction and operationalised as a low ratio of forced expiratory volume in 1 s (FEV_1_) to forced vital capacity (FVC).^2^ By far the strongest risk factors for airflow obstruction are smoking and exposure to environmental tobacco smoke,^3^but many areas of the world with high mortality rates from ‘COPD’ still have low consumption of tobacco.^4^ The distribution of death from COPD in the UK is not the same as that of lung cancer, the disease most strongly associated with tobacco consumption, but is more closely associated with low social status^5^ and poverty.^6^

A low FEV_1_ is associated with increased mortality, including a high mortality from cardiovascular disease,^7^ but there is also evidence of an association with a low FVC, a measure correlated with the FEV_1_.^8^ ^9^ When these two measures are analysed together, the high mortality is associated with the spirometric restriction and not with airflow obstruction.^10^

We examined the relation of national mortality rates from COPD, as recorded by the global health observatory, with the prevalence of airflow obstruction and spirometric restriction in 22 Burden of Obstructive Lung Disease (BOLD) study sites, and with the prevalence of smoking and poverty. We also used the BOLD data from 22 sites to describe the distribution of airflow obstruction (FEV_1_/FVC less than the lower limit of normal (<LLN)) and spirometric restriction (FVC<LLN) across these sites and their relation with smoking prevalence and poverty stratified by sex.

## Methods

The design and rationale for the BOLD study, the characteristics of samples and the prevalence of COPD in 14 sites have been reported elsewhere.^3^ ^11–13^ Data collection from an additional eight sites has been completed since these earlier publications and added to the dataset for these analyses. The study population comprised non-institutionalised people aged 40 years and older stratified by sex.

BOLD sites are selected to represent the Global Burden of Disease regions, giving greater weight to larger regions but still ensuring at least two sites in most regions. Within regions, selection of sites is largely dependent on the availability of suitable collaborators, but sites are asked to sample from substantial populations of over 250 000 from predefined administrative areas to avoid highly exceptional populations.

Response rates were defined as the number of responders (those who completed the core questionnaire and post-bronchodilator spirometry) divided by the total number of individuals contacted. Cooperation rates were defined as the number of responders divided by the total number of responders plus active refusers.

Lung function, including FEV_1_ and FVC, was measured using the ndd EasyOne Spirometer (ndd Medizintechnik AG, Zurich, Switzerland), before and 15 min after inhaling salbutamol (200 μg) from a metered dose inhaler through a spacer. Spirograms were reviewed by the BOLD Pulmonary Function Reading Centre, and assigned a quality score based on acceptability and reproducibility criteria from the American Thoracic Society (ATS) and European Respiratory Society (ERS).^14^ Spirometry technicians at BOLD sites were certified before data collection, received regular feedback on quality and were required to maintain a prespecified quality standard.

Outcome measures were airflow obstruction, defined as a post-bronchodilator FEV_1_/FVC ratio below the LLN for age and sex,^15^ based on reference equations for Caucasians derived from the third US National Health and Nutrition Examination Survey,^16^ and spirometric restriction defined as a post-bronchodilator FVC below the LLN for height, age and sex, based on the same reference population.

Information on respiratory symptoms, health status and exposure to risk factors was obtained from face-to-face interviews conducted in the subject's native language by trained and certified staff. Questions were derived from the 1978 ATS Epidemiology Standardisation Project,^17^ the European Community Respiratory Health Survey,^18^ the Consilio Nazionale Ricerche study,^19^ and the Obstructive Lung Disease in North Sweden study.^20^

National mortality data for 193 countries were obtained from the World Health Organization^21^ for two age groups, 15–59 and 60+ years, and expressed as rates/100 000 population. For each site we estimated for the age groups 40–59 years and 60+ years the prevalence of airflow obstruction and the prevalence of spirometric restriction using sampling weights to account for the sampling strategies in each site.

We compared the national COPD mortality rates against the prevalence of airflow obstruction and of spirometric restriction in the BOLD centres using Spearman rank correlation. We then regressed the same mortality rates against the gross national income (GNI) per person for the country using data from the World Bank and expressed as US dollars ($US) adjusted for purchasing power parity (PPP)^22^ and the age-standardised national prevalence of cigarette smoking obtained from the Tobacco Atlas.^23^

We finally regressed the prevalence of airflow obstruction and spirometric restriction against the mean pack years smoked in the site and against the GNI^22^ and plotted the results.

We do not expect the variance of the regression errors to be equal across observations (homoscedasticity) because the outcome and predictor variables in the regression models reflect ‘means’ of variables (rather than individual observations) from populations that vary in size. Therefore we used weighted least squares regression for which the weight is the population.^24^ The bigger the population the smaller the variance of the regression errors. In the models for national mortality data, the weight is the total number of men and women in the relevant age group in each country. In the regression models for prevalence of airflow obstruction and spirometric restriction, the weight is the total number of people with acceptable spirometry data in each site. For each model fitted residuals were plotted against the predicted values to investigate linearity of associations. When associations were not linear we transformed the predictor variable. When looking at the relationships between national mortality levels from COPD and GNI we used log GNI as the predictor variable whereas when looking at the relationship between spirometric restriction and GNI, we used 1/GNI.

To test the robustness of our findings to missing data, we reran the analyses involving data from the BOLD sites after excluding those sites that had a cooperation rate of less than 60%.

All analyses were done using Stata V.12 (Stata Corporation, College Station, Texas, USA). The correlation coefficients quoted are Spearman's rank correlation coefficients (r_s_). If appropriate, robust SEs were computed to take account of any clustering within countries. Ethical approval was obtained by each site from the local ethical committee and written informed consent was obtained from every participant.

## Results

The sampling design used at each BOLD site is presented in online supplementary table E1 together with information on response and cooperation rates at each site. A third of the sites had cooperation rates above 80%, a third between 60% and 79% and a third below 60%. Low and middle income countries and Nordic countries had the highest cooperation rates.

The estimated prevalence of airflow obstruction aged 40 years and over ranged among men from 5.7% (Pune, India) to 23.0% (Cape Town, South Africa), and among women from 4.2% (Nampicuan, The Philippines) to 20.7% (Uppsala, Sweden) (table 1). The prevalence of spirometric restriction was much more variable, ranging among men from 8.4% (Bergen, Norway) to 67.7% (Mumbai, India) and among women from 6.7% (Bergen, Norway) to 70.5% (Srinegar, India).

**Table 1:** Number of participants included, prevalence of airflow obstruction (FEV_1_/FVC<LLN) and low FVC (<LLN) and mean pack years smoked from the BOLD sites for men and women separately, and GNI/capita ($US PPP) and national smoking rates for men and women for countries containing BOLD sites

Site (dates of fieldwork)	N	Prevalence of airflow obstruction (% FEV_1_/FVC<LLN): men	Prevalence of low FVC (% FVC<LLN): men	Smoking (mean pack years): men	Prevalence of airflow obstruction (% FEV_1_/FVC<LLN): women	Prevalence of low FVC (% FVC<LLN): women	Smoking (mean pack years): women	National GNI/capita($US PPP)	National cigarette consumption (current prevalence %)	
									Adult men	Adult women
Guangzhou, China (2003)	473	9.3	30.0	21.5	6.3	30.5	1.0	6230	59.5	3.7
Mumbai, India (2006/8)	440	6.0	67.7	2.0	7.6	68.7	0.0	3000	27.6	1
Pune, India (2008/9)	849	5.7	63.1	1.2	6.8	70.5	0.1	3000	27.6	1
Srinagar, India (2010/11)	763	17.3	25.3	24.2	14.8	31.2	1.3	3000	27.6	1
Manila, The Philippines (2005/6)	893	15.1	62.4	18.7	4.2	62.9	2.7	3680	38.9	8.5
Nampicuan-Talugtug, The Philippines (2008/9)	722	16.9	52.7	20.6	13.5	61.0	3.3	3680	38.9	8.5
Sydney, Australia (2006/7)	541	7.6	16.1	14.1	14.1	9.4	9.9	35610	–	–
Krakow, Poland (2005)	526	14.9	10.7	23.7	12.2	9.6	7.5	17690	43.9	27.2
Tartu, Estonia (2008/10)	615	7.9	11.0	12.7	4.9	6.7	2.8	20630	49.9	27.5
Bergen, Norway (2005/6)	658	13.8	8.4	14.7	10.0	9.9	10.0	60220	33.6	30.4
Hannover, Germany (2005)	683	9.9	10.8	19.7	6.8	7.7	11.1	37540	–	–
Lisbon, Portugal (2008)	714	9.3	12.0	21.4	7.4	9.5	3.4	24060	40.6	31
London, UK (2006/7)	677	19.5	22.1	19.6	16.0	14.2	11.9	37490	–	–
Maastricht, The Netherlands (2007/9)	590	19.7	11.0	15.3	17.9	9.3	9.1	41840	38.3	30.3
Reykjavik, Iceland (2004/5)	757	9.2	14.9	13.9	13.5	10.0	9.8	30900	26.1	26.6
Salzburg, Austria (2004/5)	1258	13.4	11.0	17.1	20.7	8.2	8.4	39720	46.4	40.1
Uppsala, Sweden (2006/7)	547	10.5	10.2	12.2	8.7	10.0	8.0	40850	19.6	24.5
Adana, Turkey (2003/4)	806	19.8	13.1	27.0	9.1	15.7	4.3	14820	51.6	19.2
Lexington, USA (2005/6)	508	12.3	25.7	30.1	16.2	26.1	19.0	47280	–	–
Vancouver, Canada (2005/6)	827	14.5	8.4	15.0	12.5	8.4	9.2	38500	19	17.5
Cape Town, South Africa (2005)	847	23.0	47.6	16.1	16.9	46.1	8.7	10090	25	7.8
Sousse, Tunisia (2010/12)	661	8.6	25.1	2.1	1.8	27.2	0.0	8390	46.5	1

BOLD, Burden of Obstructive Lung Disease; FEV_1_, forced expiratory volume in 1 s; FVC, forced vital capacity; GNI, gross national income; LLN, lower limit of normal; PPP, purchasing power parity.

The national mortality rates attributed to COPD in those aged 15–59 years were strongly correlated with the local prevalence of spirometric restriction in the BOLD sites in those aged 40–59 (men: r_s_=+0.73, p=0.0001; women r_s_=+0.90, p≤0.0001) but not with the prevalence of airway obstruction (men: r_s_=+0.28, p=0.2; women: r_s_=+0.17, p=0.46). Similarly the national mortality rates attributed to COPD in those aged 60+ years were strongly correlated with the local prevalence of spirometric restriction in the BOLD sites in those over 60 years old (men: r_s_=+0.63, p=0.0022; women: r_s_=+0.37, p=0.1) but not with the prevalence of airflow obstruction (men: r_s_=+0.28; p=0.23; women: r_s_=+0.22, p=0.33).

**Figure 1:**
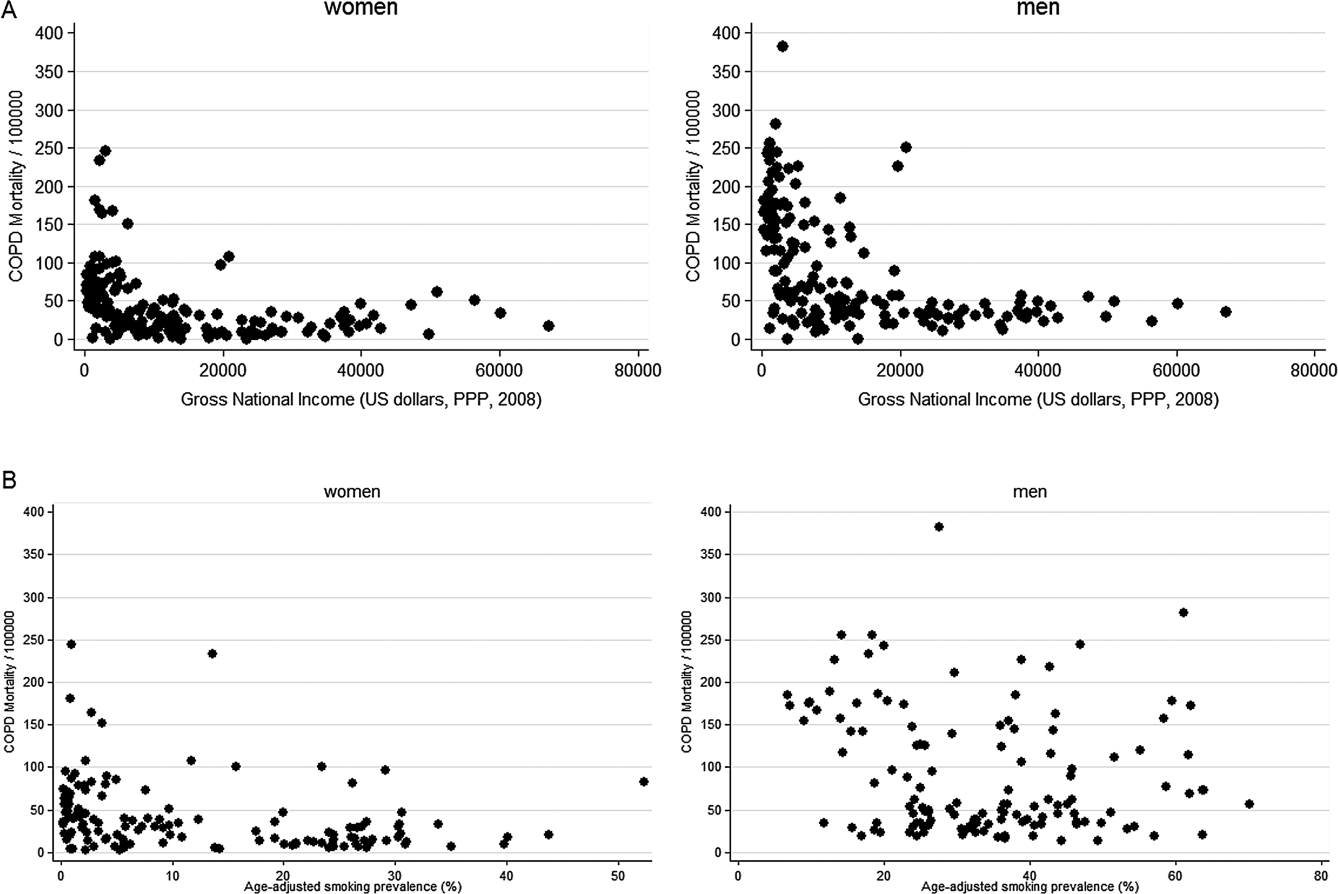
Age standardised national chronic obstructive pulmonary disease (COPD) mortality (age 15+ years) by sex and (A) annual per capita gross national income and (B) age-standardised national smoking prevalence. PPP, purchasing power parity.

Plots of COPD mortality rates for the 179 countries with available data showed a strong inverse association with GNI, with rates rising rapidly as GNI fell below US$15 000 per capita per annum (figure 1A) and showed no clear positive association with the age standardised prevalence of cigarette smoking in the 135 countries with available data (figure 1B).

Regression of national mortality rates from COPD against the logarithm of the GNI showed a strong negative association for both sexes and for both age groups (15–59 years and 60+ years) (p<0.001 in all cases) (table 2). For the 19 countries with BOLD data this association was qualitatively similar, though the regression coefficients were stronger (table 2).

**Table 2:** Association of national mortality levels from COPD with logarithm of GNI/capita ($US PPP) for all 179 countries with information and for the 19 countries with BOLD sites, and age-adjusted national smoking prevalence for 135 available countries and with mean pack years smoked for 22 BOLD sites

	Men			Women		
	β	95% CI	p Value	β	95% CI	p Value
*GNI*						
Age 15–59 years						
Log GNI (N=179)	−4.64	−5.58 to −3.69	<0.001	−2.87	−3.55 to −2.20	<0.001
Log GNI (BOLD countries: N=19)	−7.90	−11.24 to −4.55	<0.001	−4.91	−7.04 to −2.78	<0.001
Age >60 years						
Log GNI (N=179)	−196	−235 to −158	<0.001	−144	−179 to −109	<0.001
Log GNI (BOLD countries: N=19)	−329	−388 to −270	<0.001	−259	−326 to −192	<0.001
*Smoking*						
Age 15–59 years						
Age-adjusted smoking prevalence (%) (N=135)	−0.31	−0.41 to −0.22	<0.001	−0.51	−0.63 to −0.39	<0.001
Mean pack years (BOLD sites: N=22)	−0.52	−0.98 to −0.07	0.027	−0.74	−1.34 to −0.14	0.019
Age >60 years						
Age-adjusted smoking prevalence (%) (N=135)	−5.57	−9.987 to −1.164	0.014	−24.47	−28.59 to −20.36	<0.001
Mean pack years (BOLD sites: N=22)	−18.5	−31.4 to −5.5	0.008	−39.0	−58.0 to −19.9	<0.001

BOLD, Burden of Obstructive Lung Disease; COPD, chronic obstructive pulmonary disease; GNI, gross national income; PPP, purchasing power parity.

Table 2 also shows the results of regressing mortality rates from COPD against national smoking rates. Coefficients are significantly negative for both age groups and both sexes. When this analysis was repeated using the local estimates of mean cumulative pack years smoked by the whole population in the 22 BOLD sites there was also a significant negative association in all groups with more smoking being associated with a lower national mortality rate for COPD (table 2).

**Figure 2:**
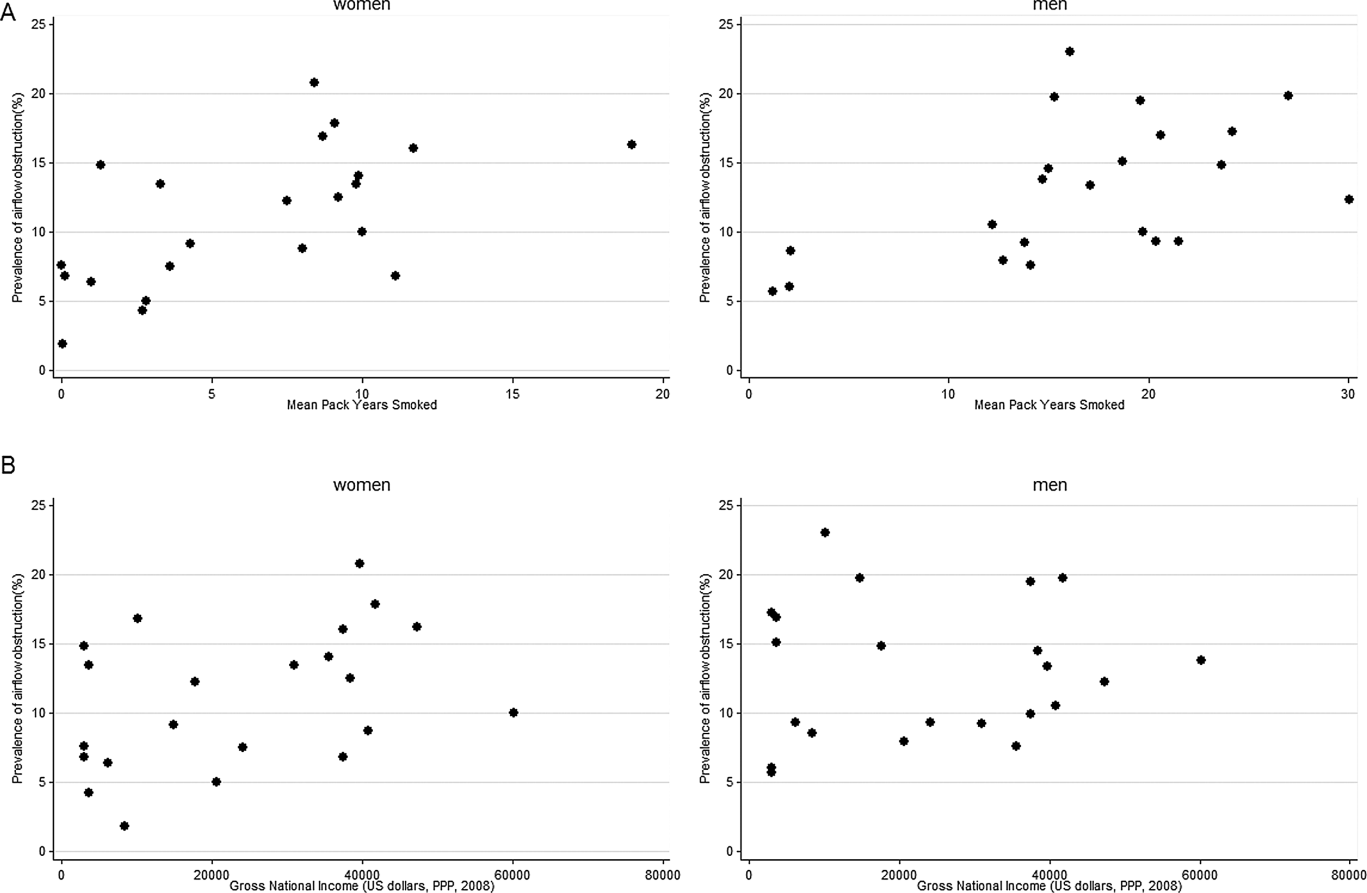
Prevalence of airflow obstruction (FEV_1_/FVC<LLN) by sex and (A) mean pack years smoked and (B) annual per capita gross national income. FEV_1_, forced expiratory volume in 1 s; FVC, forced vital capacity; LLN, lower limit of normal; PPP, purchasing power parity.

Although mortality from COPD was negatively associated with smoking, whether measured as the national age-standardised prevalence or as the local mean cumulative pack years smoked, there was a clear positive association between the prevalence of airflow obstruction and the mean pack years smoked in the 22 BOLD sites (figure 2A) accounting for 37% of the variance in men (p=0.003) and 35% of the variance in women (p=0.004) (table 3). The prevalence of airflow obstruction increased by 4.0% per 10 pack years smoked in men and by 6.7% per 10 pack years in women. The association between prevalence of airflow obstruction and GNI was weakly positive but not significant (men: p=0.78; women p=0.06) (figure 2B; table 3).

**Table 3:** Association of prevalence of airflow obstruction (%FEV_1_/FVC<LLN), spirometric restriction (% FVC <LLN) with mean pack years smoked and GNI/capita ($US PPP) in 22 BOLD sites

	Men			Women		
	β	95% CI	p Value	β	95% CI	p Value
Airflow obstruction						
Mean pack years	0.40	0.15 to 0.64	0.003	0.67	0.24 to 1.10	0.004
GNI (per US$1000 PPP)	0.02	−0.10 to 0.13	0.78	0.12	−0.01 to 0.25	0.063
Spirometric restriction						
Mean pack years	−1.07	−2.24 to 0.10	0.071	−2.32	−4.25 to −0.40	0.021
1/GNI (per US$1000 PPP)	140	101 to 179	<0.001	168	115 to 221	<0.001

BOLD, Burden of Obstructive Lung Disease; FEV_1_, forced expiratory volume in 1 s; FVC, forced vital capacity; GNI, gross national income; LLN, lower limit of normal; PPP, purchasing power parity.

**Figure 3:**
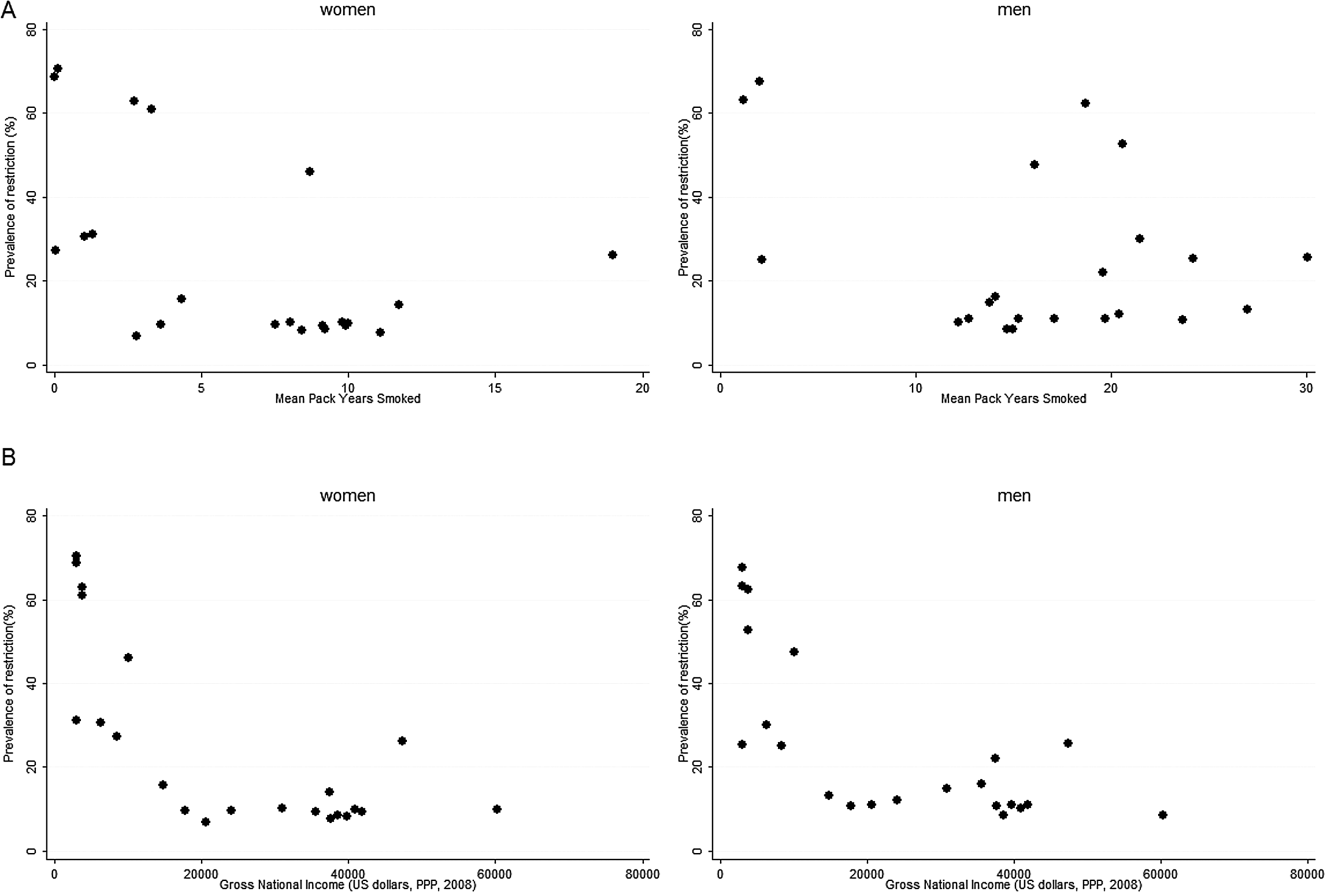
Prevalence of a spirometric restriction (FVC<LLN) by (A) mean pack years smoked and (B) annual per capita gross national income. FVC, forced vital capacity; LLN, lower limit of normal; PPP, purchasing power parity.

Table 3 also shows that spirometric restriction was slightly more common when smoking rates were higher, an association that was statistically significant for women (p=0.021) but not for men (p=0.071) (figure 3A; table 3). There was, however, a strong association between the prevalence of spirometric restriction and a lower GNI (men p<0001; women p<0.001) with rates rising rapidly as GNI fell below US$15 000/capita/annum (table 3; figure 3B).

When we reran the analyses reported in tables 2 and 3 after excluding the BOLD sites that had a cooperation rate of less than 60% the associations that we found were qualitatively similar to those using all the sites (see online supplementary tables E2 and E3).

## Discussion

The poor correlation between mortality rates from COPD and the prevalence of smoking has been remarked on before at the national^6^ and international level,^4^ ^25^ but the findings from BOLD go further in showing that smoking is a good predictor of the prevalence of airflow obstruction. Other causes of airflow obstruction, such as biomass exposure, are not therefore required to explain any discrepancy between the prevalence of obstruction and the prevalence of smoking.^4^

The strong relation of COPD mortality with poverty has also been described at a national level,^6^ and in England and Wales the social class gradient for COPD mortality is steeper than for either lung cancer or even tuberculosis.^5^ The implications for global health, however, have not been widely understood. Nor has the similarity of the global distributions of COPD mortality and the prevalence of spirometric restriction or their relation to the per capita GNI.

The prevalence of airflow obstruction correlates well with the mean pack years smoked in the BOLD sites and with national estimates of the prevalence of smoking. However, the national mortality rates from ‘COPD’ do not correlate with either the national prevalence of smoking or with the local prevalence of airflow obstruction. However, they do correlate well with the prevalence of spirometric restriction. Both ‘COPD’ mortality rates and the prevalence of spirometric restriction are strongly associated with the GNI per head of population, each rising steeply as GNI/capita falls below US$15 000 PPP.

Although we do not yet have direct evidence from the BOLD study, evidence from the USA shows that the FVC is a much stronger determinant of survival than the FEV_1_/FVC ratio.^10^ In population surveys it is unlikely that spirometric restriction represents severe obstruction, as it may do in clinics in which gas trapping due to obstruction may reduce the FVC. First, we found no association between the prevalence of airflow obstruction and spirometric restriction; second, in younger adults the FVC relates well to the total lung capacity;^26^ third, there is evidence among older people that the total lung capacity is also a good predictor of mortality and use of services;^27^ and finally, the BOLD sites with spirometric restriction are not those with a high prevalence of obstruction.

‘COPD’ is defined in terms of chronic airflow limitation^2^ but this definition has its limitations^28^ and in surveys the association between self-reported ‘COPD’ and airflow obstruction is weak.^29^ Given that very few people will have had spirometry, this discrepancy is unsurprising. In addition, given the difficulty in ante-mortem diagnosis, it is understandable if those with spirometric restriction and without airflow obstruction are certified as having died of ‘COPD’.

The BOLD project is the largest and most ambitious attempt to date to quantify the global variation in ventilatory function with associated symptoms and risk factors. The quality of lung function data from the BOLD study is controlled at a high level. All technicians were trained according to a common protocol and all the spirometric tracings were checked centrally for quality during the fieldwork and errors fed back to the technicians. When readings were inadequate, technicians were suspended until they had been retrained. Spirometry that did not reach ATS/ERS standards was rejected. Estimates of deaths by cause were taken from the Global Health Observatory of the World Health Organization^21^ and were calculated using methods summarised elsewhere.^30^

In some BOLD sites the estimates of prevalence are based on studies with relatively low response rates, but when we omitted all sites with a cooperation rate below 60%, the results did not materially change. There are very few data missing from national sources and those that are missing are generally from small countries. The main exceptions are a small number of large countries, such as the USA, Germany and Australia with missing smoking data. This small number of missing units is unlikely to make any difference to the results.

In comparing data from the BOLD sites with national data on smoking, income or mortality we are making an assumption that the sites are representative of their country. Formally this is not the case, but the assumption is nevertheless not unreasonable. First, although the sites were not randomly selected from all possible sites, we took care to sample from well defined and relatively large populations to avoid very special groups. Second, when we compare the BOLD data and the national data for smoking prevalence, we get very similar results. Third we know that the within-country variation in mortality is much less than the between-country variation, even in relatively homogeneous regions such as Europe.^31^ Finally, although in principle a misclassification arising from this assumption could explain a lack of association, it is unlikely to explain a strong association such as that between the prevalence of spirometric restriction and GNI.

The lack of association between smoking prevalence and mortality from ‘COPD’ is explained by the inverse association between poverty and smoking prevalence. At an individual level there is strong evidence from the BOLD study for an association between airflow obstruction and smoking,^12^ an association also reflected in the BOLD study at an ecological level (figure 2A). At an ecological level, however, there is no association between smoking and mortality from ‘COPD’.

We can only speculate about the reasons that FVC is low in poor countries. In some countries different norms are recommended for different ethnic groups^16^ ^32^ and there is a common belief that FVC is racially determined, though the evidence for this is weak.^33^ In the UK BOLD study, African Caribbean and Asian, mostly South Asian, participants had similarly reduced FVC,^34^ and in the current analysis the associations with poverty are even stronger when the predominantly white European populations are excluded. The strongest association is seen in countries where the GNI is less than US$15 000 per annum and contains an ethnically very diverse group of communities, including populations in India, the Philippines, China, Tunisia and Turkey, and a Cape coloured community in South Africa which has a mixed Xhosa, Khoi, European and Malay ancestry. It is unlikely that genetics could explain away the strong association between spirometric restriction and poverty in this population. The observed coincidence of the high prevalence of spirometric restriction and the high mortality rate from ‘COPD’ is consistent with the earlier finding that the prognostic significance of a given FVC is independent of ethnicity and supports our decision not to adjust the lower limits of normal for ethnicity.^35^

The high prevalence of spirometric restriction in low-income countries is likely to be largely due to unknown environmental causes. There is a strong association with poverty but it is important to understand how this is mediated. Low birth weight is associated with spirometric restriction in many studies,^26^ ^36–39^ and low birth weight is more common in developing countries (16%) and in the least developed countries (17%) when compared with industrialised countries (7%).^40^ Specific exposures also associated with spirometric restriction include exposure to indoor air pollution^41^ and a poor diet.^42^ More speculative risk factors include early infections^43^ and early exposure to biomass fuel.^44^

The mechanism by which spirometric restriction leads to death is also unclear, but it is unlikely that the spirometric restriction represents a high prevalence of classical restrictive lung diseases as these conditions are rare. Low ventilatory function is associated with other comorbidities and the excess deaths among those with low ventilatory function are often ascribed to cardiovascular causes.^7^ Comorbidities could explain some of the association between low lung volumes, measured as spirometric restriction or FEV_1_, and increased mortality.^7^ Nevertheless the fact that the deaths are ascribed to ‘COPD’ suggests that they are associated with substantial respiratory symptoms.

Tobacco is the highest ranked risk factor for disease burden in high-income North America and Western Europe and second only to high blood pressure globally according to the most recent estimates.^45^ Nevertheless, in low-income countries other factors associated with poverty dominate the risk of mortality attributed to ‘COPD’, even though spirometrically measured chronic airflow obstruction remains overwhelmingly a condition associated with smoking in all regions. There is a serious danger that an epidemic of smoking, if ever it were to become established in these vulnerable regions, would have even more devastating effects than we have seen so far in the more affluent countries.

It is unlikely that the high mortality attributed to ‘COPD’, particularly in low-income countries, is associated with chronic airflow obstruction. It is much more likely to be associated with spirometric restriction. These analyses challenge us to rethink our notions and beliefs about the origins and significance of chronic lung disease and its prominent role as a major cause of death in low-income countries. This by no means reduces the importance of tobacco control as the most important approach to the prevention of chronic airflow obstruction and other morbidity.

## Supplementary Material

Web supplement
