# Supplementary material for: Chronic obstructive pulmonary disease mortality and prevalence: the associations with smoking and poverty—a BOLD analysis
Source: Thorax. 2013 Dec 18;69(5):465–73. doi: 10.1136/thoraxjnl-2013-204460 (PMC3995258; doi:10.1136/thoraxjnl-2013-204460)
Supplement: Web supplement [file thoraxjnl-2013-204460-s1.pdf]

Supplementary Table E1. Sampling design and participation rates by BOLD site

Site	Sampling design	N	Response rate(%)	Cooperation rate (%)
Guangzhou, China	Stratified random sample	602	87	87
Adana, Turkey	Stratified cluster sample	875	82	85
Salzburg, Austria	Stratified random sample	1349	65	67
Capetown, South Africa	Cluster sample	896	63	68
Reykjavik, Iceland	Simple random sample	758	81	84
Hannover, Germany	Stratified random sample	713	59	61
Krakow, Poland	Stratified random sample	603	78	79
Bergen, Norway	Stratified random sample	707	68	71
Vancouver, Canada	Random digit dialling	856	26	51
Manila, Philippines	Stratified cluster sample	918	58	58
Lexington, USA	Random digit dialling	563	14	27
Sydney, Australia	Stratified random sample	585	25	33
London, England	Stratified random sample	697	17	37
Uppsala, Sweden	Stratified random sample	588	61	63
Mumbai, India	Stratified cluster sample	515	55	66
Lisbon, Portugal	Stratified cluster sample	745	10	27
Maastricht, The Netherlands	Stratified random sample	634	48	55
Nampicuan & Talugtug , Philippines	Stratified cluster sample	991	85.5	86.2
Tartu, Estonia	Stratified random sample	658	49	70
Pune, India	Simple random sample	1388	97	97
Sousse, Tunisia	Stratified cluster sample	717	90	92
Srinagar, India	Stratified cluster sample	953	86.9	88

N – Total number of responders: defined as participants who completed the core questionnaire and have post-bronchodilator spirometry

Supplementary TABLE E2: Association of National Mortality levels from COPD with logarithm of Gross National Income / capita ($US PPP) for the 13 countries with BOLD sites (where cooperation rate was more than 60%) and with mean pack years smoked for 15 BOLD sites (where cooperation rate was more than 60%).

	Men			Women		
	β	95% CI	p	β	95% CI	p
Age 15-59 years						
Mean Pack Years (BOLD sites: N=15)	-0.45	-1.05, 0.14	0.122	-1.72	-2.61, -0.85	0.001
Log GNI (BOLD countries: N=13)	-13	-21.75, -4.25	0.007	-8.29	-13.41, -3.16	0.004
Age > 60 years						
Mean Pack Years (BOLD sites: N=15)	-11.8	-21.3, -2.3	0.019	-76.9	-95.4, -58.3	<0.001
Log GNI (BOLD countries: N=13)	-383	-445, -320	<0.001	-301	-372, -230	<0.001

Supplementary TABLE E3. Association of prevalence of airflow obstruction (%FEV1/FVC < LLN) ,spirometric restriction (%FVC < LLN) with mean pack years smoked and Gross National Income / capita ($US PPP) in 15 BOLD sites where cooperation rate was more than 60%.

	Men			Women		
	β	95%CI	p-value	β	95%CI	p-value
**Airflow obstruction**						
Mean Pack Years	0.45	0.20, 0.71	0.002	0.66	-0.05, 1.38	0.067
GNI (per $1000PPP)	-0.01	-0.13, 0.12	0.92	0.06	-0.13, 0.25	0.514
**Spirometric restriction**						
Mean Pack Years	-1.32	-2.63, -0.02	0.048	-3.09	-.5.88, -0.31	0.032
1/GNI (per $1000PPP)	134	110, 159	<0.001	158	113, 204	<0.001
